# Global Transcriptomic Changes Induced by Infection of Cucumber (*Cucumis sativus* L.) with Mild and Severe Variants of Hop Stunt Viroid

**DOI:** 10.3389/fmicb.2017.02427

**Published:** 2017-12-12

**Authors:** Changjian Xia, Shifang Li, Wanying Hou, Zaifeng Fan, Hong Xiao, Meiguang Lu, Teruo Sano, Zhixiang Zhang

**Affiliations:** ^1^State Key Laboratory for Biology of Plant Diseases and Insect Pests, Institute of Plant Protection, Chinese Academy of Agricultural Sciences, Beijing, China; ^2^State Key Laboratory for Agro-Biotechnology, Key Laboratory of Pest Monitoring and Green Management, Department of Plant Pathology, College of Plant Protection, China Agricultural University, Beijing, China; ^3^Key Laboratory of Tobacco Pest Monitoring Controlling and Integrated Management, State Tobacco Monopoly Bureau, Institue of Tobacco Research, Chinese Academy of Agricultural Sciences, Qingdao, China; ^4^Faculty of Agriculture and Life Science, Hirosaki University, Hirosaki, Japan

**Keywords:** hop stunt viroid, cucumber, RNA-seq, *RNA-dependent RNA polymerase 1 (CsRDR1)*, salicylic acid, photosynthesis

## Abstract

Fifteen years after transfer to hops, hop stunt viroid-grapevine (HSVd-g) was replaced by HSVd-hop (HSVd-h), a sequence variant that contains changes at five different positions. HSVd-g54 is a laboratory mutant derived from HSVd-g that differs from its progenitor by a single G to A substitution at position 54. While infection by HSVd-h induces only mild stunting in cucumber (*Cucumis sativus* L.), HSVd-g54 induces much more severe symptoms in this indicator host. Comparison of transcriptome profiles of cucumber infected with HSVd-h or HSVd-g54 with those of mock-inoculated controls obtained by whole transcriptome shotgun sequencing revealed that many genes related to photosynthesis were down-regulated following infection. In contrast, genes encoding RNA-dependent RNA polymerase 1 (*CsRDR1*), especially *CsRDR1c1* and *CsRDR1c2*, as well as those related to basal defense responses were up-regulated. Expression of genes associated with phytohormone signaling pathways were also altered, indicating that viroid infection initiates a complex array of changes in the host transcriptome. HSVd-g54 induced an earlier and stronger response than HSVd-h, and further examination of these differences will contribute to a better understanding of the mechanisms that determine viroid pathogenicity.

## Introduction

Viroids are small, circular, non-protein-coding infectious RNAs that have the ability to replicate autonomously when inoculated into higher plants. Their genomic RNAs range in size from 246 to 401 nucleotides (nt) (Flores et al., 2015; Daròs, 2016; Gago-Zachert, 2016), and some viroids infect economically important crops where they can cause devastating diseases. The symptoms of viroid diseases include chlorosis, epinasty, leaf deformation and necrosis, stunting, fruit distortion, and plant death (Flores et al., 2005). As demonstrated for several other types of plant pathogens (Boyko et al., 2007; Babu et al., 2008), comprehensive analysis of gene expression in viroid-infected host is critical to understanding the molecular mechanisms responsible for viroid pathogenesis.

Published studies have used either mRNA differential display (Tessitori et al., 2007) or hybridization-based microarray technologies (Owens et al., 2012; Rizza et al., 2012) to examine the number and types of host genes that are differentially expressed in response to viroid infection. The viroid-host combination most frequently studied has been tomato plants infected with potato spindle tuber viroid (PSTVd). Among the biological functions affected by viroid infection are defense and stress responses, cell wall structure, chloroplast biogenesis, protein metabolism, peroxidase, and symporter activities (Itaya et al., 2002; Owens et al., 2012; Rizza et al., 2012). Genes involved in the biosynthesis of gibberellin, jasmonic acid, and other phytohormones also showed changes in expression during PSTVd infection (Wang et al., 2011; Owens et al., 2012). It should be noted that most of the host genes whose expression was altered by viroid infections are sensitive to many different biotic stresses, indicating that one or more shared signal transduction pathways or gene regulation networks may be involved in viroid disease induction. However, there is not enough data at present to construct a clear pathway or network to explain viroid disease progression. A recently-developed large-scale RNA sequencing-based strategy (i.e., RNA-seq; Wang et al., 2009) promises to change this situation.

RNA-seq is a high-throughput approach to transcriptome profiling that uses next generation sequencing (NGS) technologies for the analysis of gene expression (Wang et al., 2009; Ozsolak and Milos, 2011). RNA-seq eliminates several of the problems associated with microarray technologies, including its restriction to known genes and limited dynamic range of detection (Ozsolak and Milos, 2011; Van Verk et al., 2013). RNA-seq can generate a far more precise estimate of mRNA levels and transcript isoforms at a much lower cost. These advantages, coupled with higher reproducibility and lower RNA sample requirements (Wang et al., 2009; Van Verk et al., 2013) make RNA-seq a powerful tool for global genome expression studies. It can be used to investigate specific developmental stages or physiological conditions as well as tissues in normal or diseased states (Ozsolak and Milos, 2011; Van Verk et al., 2013).

In plant pathology, transcriptome analysis has been widely used to examine global gene expression profiles in plants infected with fungi, bacteria, or virus. However, only three such studies have been performed for viroid-infected plants. Most recently, the transcriptome of PSTVd-infected tomato (Zheng et al., 2017), hop stunt viroid (HSVd)-infected hop (Kappagantu et al., 2017) and hop latent viroid (HLVd)- and citrus bark cracking viroid (CBCVd)-infected hop (Pokorn et al., 2017) were comprehensively analyzed. Tomato genes involved in plant immune responses, mainly those in the calcium-dependent protein kinase and mitogen-activated protein kinase signaling cascades were found to be strongly up-regulated in response to PSTVd infection (Zheng et al., 2017). Hop genes involved in defense, lipid and terpenoid metabolism showed differential expression due to HSVd infection (Kappagantu et al., 2017). Many hop transcripts share nucleotide similarities with HLVd- and CBCVd-derived small RNAs, so they could be silenced in an RNA interference process, and four pathogenesis related genes were highly expressed in leaves of HLVd- and CBCVd-infected hop plants (Pokorn et al., 2017). These results provide new insights into viroid pathogenesis. Comparable analyses of other viroid-host combinations should be helpful for understanding the mechanisms that underlie viroid disease induction.

In this study, we employed HSVd-infected cucumber (*Cucumis sativus* cv. “Suyo”) as a model system in which to examine genome-wide changes in gene expression using RNA-seq. Cucumber plants infected by two HSVd variants that differ in pathogenicity were analyzed at different times post inoculation. We found that the severe variant (HSVd-g54) induced an earlier and stronger response than the mild variant (HSVd-h); furthermore, HSVd infection depressed photosynthesis, disrupted phytohormone homeostasis, and possibly induced the accumulation of salicylic acid (SA). HSVd infection also triggered basal defense responses and the expression of genes coding for RNA-dependent RNA polymerase 1 (*CsRDR1*), specially *CsRDR1c1* and *CsRDR1c2*. These results provide insights into the interaction between cucumber and HSVd and contribute to a better understanding of viroid pathogenesis.

## Materials and methods

### HSVd variants

Two different variants of HSVd were used in our experiments. Both HSVd-h (AB039271) and HSVd-g54 are related to an HSVd isolate (AB219944) originally retrieved from grapevine in Japan, HSVd-g (grapevine) (Sano et al., 2001). As described by Kawaguchi-Ito et al. (2009), HSVd-h is a novel variant recovered from hop plants inoculated with HSVd-g 15 years earlier; it differs from HSVd-g at five positions. HSVd-g54, in contrast, contains only a single A/G substitution at position 54 with respect to HSVd-g.

### Preparation of inoculum

Infectious clones of HSVd-h and HSVd-g54 were constructed by overnight incubation of unit-length cDNAs with blunt ends with T4 DNA ligase (Promega) at 4°C to generate dimers. Dimeric cDNAs were purified by gel electrophoresis, a dA was added to the 3′ end using *Taq* DNA polymerase (Tiangen Biotech, Beijing), and the modified dimers were ligated into the pGEM-T vector (Promega). Two recombinant plasmids containing head-to-tail tandem cDNA repeats, pG-dHSh and pG-dHSg54, were then selected by DNA sequencing.

Because the HSVd-h cDNA dimer was ligated into the vector in the forward orientation, pG-dHSh was linearized with *Spe* I (Takara) before *in vitro* transcription using T7 RNA polymerase (Promega). HSVd-g54 cDNA dimer was ligated into the vector in reverse orientation; thus, the resulting recombinant plasmid was linearized with *Nco* I (Takara) before transcription using SP6 RNA polymerase (Promega). Before inoculation RNA transcripts were adjusted to a final concentration of 100 ng RNA/μL in 100 mM sodium phosphate buffer (pH 7.5) containing 0.3 mg/ml bentonite.

### Plant growth and inoculation

Cucumber seedlings were grown in a greenhouse with supplementary lighting (16 h per day). The temperature was maintained at 28°C in the daytime and 25°C at night. One-week-old seedlings with two expanded cotyledons were used for viroid inoculation. One cotyledon of each seedling was dusted with carborundum (600 mesh) and rubbed with 20 μL aliquots of inoculum using a sterile cotton swab. Twelve cucumber seedlings were inoculated for each treatment (HSVd-g54, HSVd-h and mock). Inoculated plants were rinsed with distilled water, placed in darkness for 8 h, and then kept in the greenhouse with the same environmental conditions for 4–5 weeks.

### RNA extraction, RT-PCR, cloning, and sequencing

Total RNAs used for northern-blot hybridization and RT-PCR detection were extracted from cucumber cotyledons (2 dpi) and top leaves (14 and 28 dpi) using a CTAB method as described previously (Adkar-Purushothama et al., 2015). For RNA-seq and RT-qPCR analysis, total RNAs from cotyledons (2 dpi) and top leaves (14 and 28 dpi) of the inoculated cucumber plants were extracted using TRNzol-A+ reagent according to the manufacturer's instructions (Tiangen Biotech, Beijing). The quality and quantity of all RNA samples were assessed by agarose gel electrophoresis and absorbance at 260 nm using a NanoDrop 2000 spectrophotometer (ThermoFisher Scientific). Furthermore, the integrity of the RNA preparations used for cDNA libraries for RNA-seq was evaluated using an Agilent Technologies 2100 bioanalyzer.

Reverse transcription (RT) was performed using M-MLV reverse transcriptase (Promega) with the HSVd-specific primer HSV-105M (5′-GCTGGATTCTGAGAAGAGTT-3′, complementary to HSVd residues 105-86), at 42°C for 1 h. The genome of HSVd was amplified by high-fidelity *Pfu* DNA polymerase (Tiangen Biotech, Beijing) using the primer pair HSV-78P (5′-AACCCGGGGCAACTCTTCTC-3′, homologous to HSVd residues 78-95) and HSV-83 M (5′-AACCCGGGGCTCCTTTCTCA-3′, complementary to HSVd residues 83-66) as reported previously (Sano et al., 2001). The PCR products were cloned into the pTOPO-Blunt Vector in the Zero Background pTOPO-Blunt Cloning Kit (Aidlab, Beijing) as indicated by the manufacturer's instructions. The recombinant plasmids were transformed into *Escherichia coli* DH5α cells, and positive clones were randomly selected for sequencing.

### Northern-blot hybridization

Equal amounts of total RNA (2 μg) from each sample were subjected to agarose gel electrophoresis. The fractionated RNAs in the gel were transferred to Hybond N+ nylon membranes using a vacuum transfer system (Bio-Rad) in 2 × SSC buffer. Northern-blot hybridization was performed at 68°C overnight with HSVd-specific full length probes that were synthesized with a DIG RNA labeling kit (Roche) according to the manufacturer's instructions. Immunological detection was performed by incubating the membrane with the chemiluminescent substrate and subsequent exposure to X-ray film (Kodak).

### Library construction and RNA-seq analysis

Libraries for RNA-seq were constructed from 3 μg samples of RNA using the NEBNext® Ultra™ RNA Library Prep Kit for Illumina® (NEB) according to the supplied instruction manual. Different index codes were added to distinguish the libraries prepared from each sample. In brief, mRNA was purified from total RNA using poly-T attached to magnetic beads and then fragmented using divalent cations at high temperature in NEBNext First Strand Synthesis Reaction Buffer (5×). First-strand cDNA was synthesized with M-MLV reverse transcriptase (RNase H-) (Promega) using random hexamer primers; second-strand cDNA was synthesized using DNA polymerase I (Promega). The remaining overhangs of the double-stranded cDNA were converted into blunt ends using the exonuclease/polymerase activities of DNA polymerase (Promega). After adenylation of the 3′ termini, the cDNA fragments were ligated to NEBNext adaptors containing a hairpin loop structure. The resulting adaptor-ligated cDNA fragments (150–200 bp) were purified using the AMPure XP system (Beckman Coulter). The size-selected, adaptor-ligated cDNA was then incubated with 3 μL of USER Enzyme (NEB) at 37°C for 15 min followed by 5 min at 95°C. PCR amplification was performed using Phusion High-Fidelity DNA polymerase (NEB) with universal primers and an index (X) primer. Finally, the PCR products purified using the AMPure XP system were run on the Agilent Bioanalyzer 2100 to assess library quality. DNA sequencing was performed by Novogene (Beijing) on an Illumina HiSeq 2500 sequencing instrument.

### Identification of differentially expressed genes

The DESeq R package (1.18.0) (Anders and Huber, 2010, 2013; Wang et al., 2010a) was used to compare the expression levels of host genes between the different samples. This software provides statistical routines for determining differential expression from digital gene expression (DGE) data using a model based on the negative binomial distribution; the resulting *P*-values were adjusted using the Benjamini and Hochberg approach (Benjamini and Hochberg, 1995) for controlling the false discovery rate (FDR). Those with an adjusted *P*-values < 0.05 were considered as differentially-expressed genes.

### GO and KEGG enrichment for differentially expressed genes

Gene Ontology (GO) enrichment analysis of differentially-expressed genes was carried out using the GOseq R package (Young et al., 2010), which corrects for gene length bias. GO terms with corrected *P*-values < 0.05 were considered to be significantly enriched.

We applied the KEGG database (http://www.genome.jp/kegg/) for understanding high-level functions and utilities of a biological system, such as the cell, the organism, and the ecosystem, from molecular-level information, especially large-scale molecular datasets generated by genome sequencing and other high-throughput experimental technologies. We used the KOBAS software (Mao et al., 2005) to search for statistical enrichment of differentially-expressed genes in KEGG pathways. KEGG terms with corrected *P*-values < 0.05 were considered to be significantly enriched in differentially-expressed genes.

### RT-qPCR

RT-qPCR was used to test the accumulation of HSVd in HSVd-infected cucumber plants and to validate the expression levels of cucumber genes in those samples analyzed by RNA-seq. Equal amounts of total RNA (2 μg) from each sample were used for cDNA synthesis by M-MLV reverse transcriptase (Promega) with random hexamer primers (Takara). The primers used for qPCR were designed using Integrated DNA Technologies (IDT) SciTools® Web Tools (PrimerQuest® program, IDT, Coralville, USA, http://www.idtdna.com/Scitools) except those for *CsRDR1a, CsRDR1b, CsRDR1c1* (Leibman et al., 2017), *CsPR1* (Kuzniak et al., 2015), and *CsEF1*α (Wan et al., 2010) genes (Table S1). qPCR was performed on a MyGo Pro Real-Time PCR System (IT-IS Life Science Ltd., UK) using GoTaq® qPCR Master Mix containing SYBR® Green I (Promega) as instructed. The cucumber *EF1*α gene was used as an internal reference gene for normalization of expression levels (Wan et al., 2010).

### Photosynthetic rate measurements

The photosynthetic rates of cucumber plants were measured with an LI-6400 Portable Photosynthesis System (LI-COR Biosciences, Inc., USA) as instructed by the manufacturer. Artificial illumination was supplied to the leaf from the system red-blue LED light source, and the photosynthetic rate was monitored as follows: six plants were randomly selected from each treatment and the temperature in the sample chamber was set to (28 ± 0.5)°C. The level of photosynthetically active radiation (PAR) was then reduced in a step-wise fashion from 2,000 to 0 μmol/(m^2^·s), and measurements were made once the leaf had attained a steady state. The resulting data were normalized using the total leaf area.

## Results

### HSVd sequence variants induce different symptoms in cucumber

**Figure 1:**
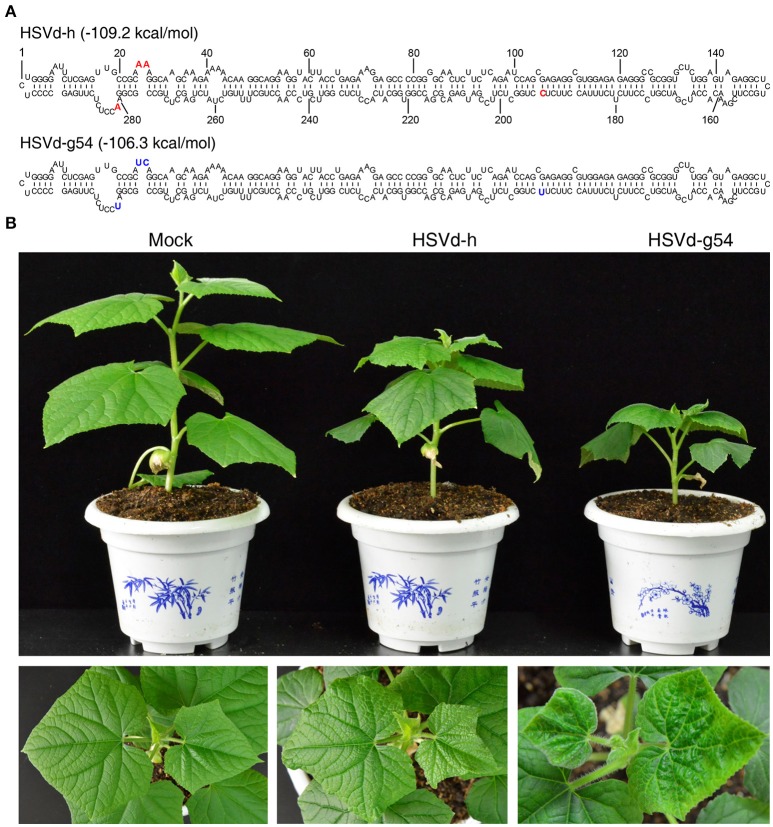
Secondary structures of hop stunt viroid variants HSVd-h and HSVd-g54, and disease symptoms of cucumber plants infected with HSVd-h and HSVd-g54. **(A)** The positions of nucleotides in HSVd-g54 that differ from HSVd-h are shown in bold type. **(B)** Differences in plant height and leaf morphology among cucumber plants inoculated with sodium phosphate buffer (mock), HSVd-h, and HSVd-g54. Note that the leaves of plants infected with HSVd-g54 were more wrinkled than those infected with HSVd-h.

**Figure 2:**
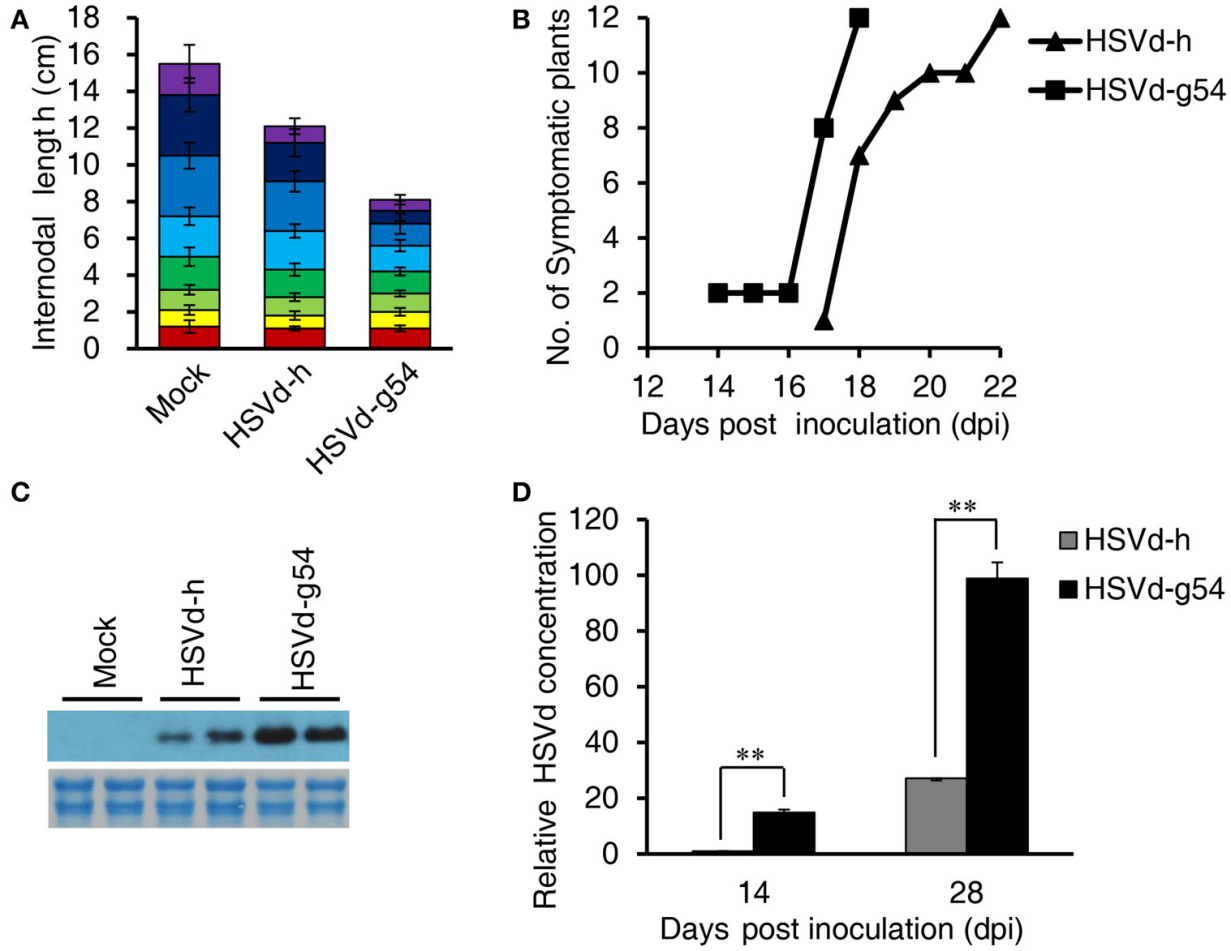
Effect of HSVd-h and HSVd-g54 infection on cucumber growth and development. **(A)** Internode lengths of cucumber plants at 28 dpi. Individual colored bars represent the lengths of successive internodes, with the first internode shown at the bottom. Measurements were taken from 12 cucumber plants per treatment, and data are expressed as the mean ± standard deviation. Error bars indicate the standard deviations of 12 replicates. **(B)** Time course of symptom appearance in cucumber plants following viroid inoculation. **(C)** Comparison of the concentrations of the two HSVd variants at 28 dpi by Northern blotting. Total RNAs (2 μg) were fractionated by electrophoresis in 1.5% (w/v) agarose gels and used for loading controls (below the hybridization signals). **(D)** Relative levels of viroid RNA as determined by RT-qPCR. Experiments were performed in triplicate and the data are expressed as the mean ± standard deviation. Error bars indicate the standard deviations of three biological replicates of RT-qPCR analysis. Double asterisks indicate statistically significant differences compared with the control at *p* < 0.01 in Student's *t-*test.

As shown in Figure 1A, there are four nucleotide differences between variants HSVd-h and HSVd-g54. When *in vitro* transcribed infectious transcripts corresponding to these two variants were inoculated into cucumber seedlings, they induced the appearance of either mild (HSVd-h) or severe (HSVd-g54) symptoms in the foliage (Figure 1B). Leaves of HSVd-h-infected plants were rough and mild wrinkled, whereas those infected with HSVd-g54 were severely wrinkled and distorted. Plants infected with HSVd-g54 also had shorter internodes than did those infected with HSVd-h (Figure 2A), resulting in reduced height of HSVd-g54-infected plants compared with those infected with HSVd-h. The timing of symptom expression was also different for these two variants. For example, all plants inoculated with HSVd-g54 displayed visible symptoms at 18 dpi, whereas those inoculated with HSVd-h displayed visible symptoms only at 22 dpi (Figure 2B). Similar results were observed in three independent experiments, confirming that HSVd-h and HSVd-g54 behave as mild and severe variants in cucumber, respectively.

The accumulation levels of HSVd-h and HSVd-g54 progeny in cucumber plants were compared by northern-blot hybridization and RT-qPCR. HSVd titers in HSVd-g54-infected plants were about 15- and 4-fold higher than those in HSVd-h-infected plants at 14 and 28 dpi (Figures 2C,D, Figure S1). To examine the stability of these two variants, HSVd cDNAs were amplified from two plants infected with each variant at 14 and 28 dpi. Five sequences were obtained from each sample, and comparison with the original sequences failed to reveal the presence of any mutants in HSVd-h- and HSVd-g54-infected plants.

### RNA-seq analysis of HSVd-g54- and HSVd-h-infected cucumber

To compare the interaction of HSVd-h and HSVd-g54 with cucumber at the transcriptional level, non-inoculated cotyledon at 2 dpi and top leaves at 14 and 28 dpi of cucumber plants infected with the two HSVd variants were collected for transcriptome analysis. Next generation sequencing generated 34.33–48.28 million raw reads per sample. After low quality reads and adapter sequences were removed, 33.29–46.67 million clean reads remained for each sample. Quality control parameters (Table S2) indicated that the resulting gene transcript data were reliable. In addition, the correlation of gene expression patterns and levels between biologically repeated samples was quite consistent, and *R*^2^ values were between 0.956 (between biologically repeated HSVd-g54 infected plants at 28 dpi) and 0.994 (between biologically repeated HSVd-h infected plants at 2 dpi) (Figure S2), indicating high repeatability of the sequencing.

### Comparison of gene expression in plants infected with HSVd-g54 and HSVd-h

Prior to identifying the differentially expressed genes (DEGs) associated with HSVd-g54 and HSVd-h infection in cucumber, gene expression levels in the different samples were calculated using FPKM (expected number of Fragments Per Kilobase of transcript sequence per Million base pairs sequenced) (Trapnell et al., 2010). The number of genes expressed in each sample was more than 19,000, which represents most of the genes predicted from sequence analysis of the cucumber genome (Huang et al., 2009). Expression levels of these genes in the different treatments were compared using the DEGSeq package (Anders and Huber, 2010).

**Figure 3:**
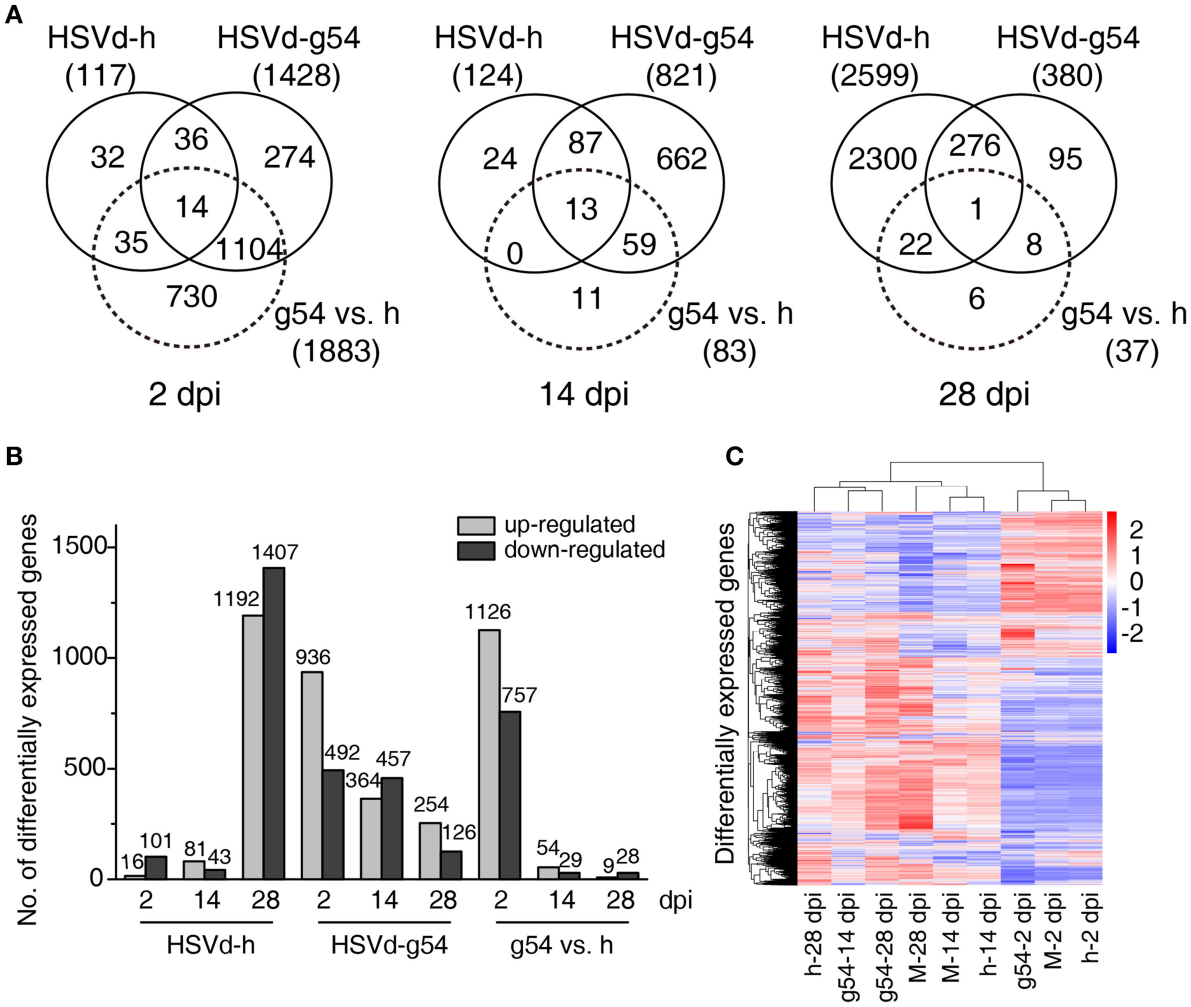
Comparison of differentially expressed genes (DEGs) in response to HSVd-g54 and HSVd-h infection of cucumber. **(A)** Venn diagrams showing unique and common DEGs in different comparisons at 2, 14, and 28 dpi. **(B)** The number of up- and down-regulated DEGs in different comparisons. **(C)** A heat cluster map of cucumber DEGs resulting from HSVd infection. Red, up-regulated genes; blue, down-regulated genes; the red and blue colors represent the values of log_10_(FPKM + 1) from high to low. M, mock infected plants; h, HSVd-h; g54, HSVd-g54.

HSVd-h and HSVd-g54 infection triggered different patterns of changes in gene expression in cucumber plants. HSVd-h induced very few DEGs at 2 and 14 dpi, but this number suddenly increased to 2599 at 28 dpi. HSVd-g54, in contrast, induced the highest number of DEGs (1428) at 2 dpi, and this number then gradually fell with time (Figure 3A, Table S3). Figure 3B compares these trends in the number of up- and down-regulated genes in cucumber plants infected with HSVd-h and HSVd-g54 in greater detail, and a heat cluster map (Figure 3C) shows that the differences in DEGs expression patterns among three treatments at 14 and 28 dpi were larger than those at 2 dpi.

**Figure 4:**
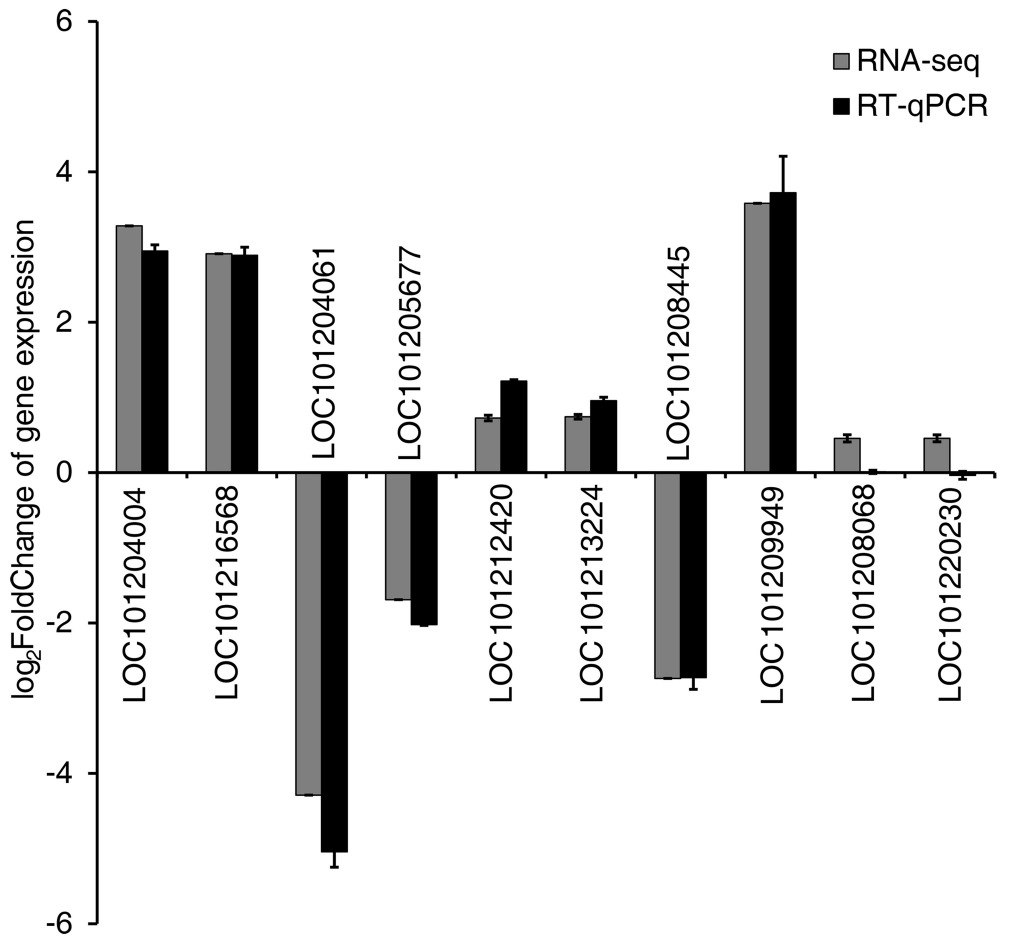
Validation of RNA-seq results by quantitative real-time PCR (RT-qPCR). Expression patterns of 10 representative genes as determined by RT-qPCR and RNA-seq. Normalization for RT-qPCR was performed using expression of the *EF1*α gene as an internal reference. Error bars on the black boxes indicate the standard deviations of three biological replicates of RT-qPCR analysis. The bars on the gray boxes indicate the false discovery rates from the two biological replicates of RNA-seq analysis.

To validate the RNA-seq results, ten genes showing different levels of expression (Table S4) were selected, and their expression levels were analyzed by RT-qPCR using gene-specific primers (Table S1). Results showed that the expression profiles of eight of these ten genes were very similar to those determined by RNA-seq (Table S4, Figure 4).

### Gene ontology enrichment analysis

To analyze the function of DEGs showing up- or down-regulation in response to HSVd-h or HSVd-g54 infection, we performed GO enrichment analysis. Individual GO terms were considered to be enriched if their corrected *P*-value was < 0.05, and Table S5 contains a list of all enriched GO terms.

Table 1 shows the top 20 enriched GO terms (all GO terms are listed if the number of enriched GO terms was <20) for the up-regulated DEGs at various time points. Note that the up-regulated DEGs are mainly enriched in protein phosphorylation, protein modification processes, protein kinase activity, phosphotransferase activity, and ribonucleotide binding in cucumber plants infected with the mild variant HSVd-h at 14 and 28 dpi. Plants infected with the severe variant HSVd-g54 showed a very similar pattern of GO terms enrichment at 2 and 14 dpi, while the four enriched GO terms at 28 dpi were very different (Table 1).

**Table 1:** The top 20 (>20) or all (≤20) enriched GO terms for the up-regulated DEGs in HSVd-infected cucumber plants.

**GO accession**	**Description**	Term type^*^	**Corrected *P*-value^†^**				
			**HSVd-h**		**HSVd-g54**		
			**14 dpi**	**28 dpi**	**2 dpi**	**14 dpi**	**28 dpi**
GO:0006468	Protein phosphorylation	BP	3.76E-04	1.84E-11	7.78E-15	4.15E-12	
GO:0016310	Phosphorylation	BP	6.95E-04	1.07E-09	6.41E-13	4.24E-11	
GO:0006464	Cellular protein modification process	BP	2.53E-03	3.71E-08	2.85E-14	1.01E-10	
GO:0036211	Protein modification process	BP	2.53E-03	3.71E-08	2.85E-14	1.01E-10	
GO:0043412	Macromolecule modification	BP	1.21E-02	6.63E-06	1.97E-11	1.15E-09	
GO:0006796	Phosphate-containing compound metabolic process	BP	3.91E-02	3.35E-07	6.04E-11	8.66E-09	
GO:0006793	Phosphorus metabolic process	BP	3.91E-02	3.35E-07	6.04E-11	8.66E-09	
GO:0044267	Cellular protein metabolic process	BP			8.55E-06	7.89E-06	
GO:0007154	Cell communication	BP		7.66E-05			
GO:0050794	Regulation of cellular process	BP					3.93E-02
GO:0050789	Regulation of biological process	BP					3.93E-02
GO:0005578	Proteinaceous extracellular matrix	CC			1.31E-08		
GO:0031012	Extracellular matrix	CC			7.76E-08		
GO:0004672	Protein kinase activity	MF	6.78E-05	4.12E-12	7.78E-15	1.25E-12	
GO:0016773	Phosphotransferase activity, alcohol group as acceptor	MF	3.76E-04	1.98E-09	1.79E-13	3.26E-11	
GO:0016301	Kinase activity	MF	6.95E-04	9.20E-09	1.24E-12	8.76E-10	
GO:0016772	Transferase activity, transferring phosphorus-containing groups	MF	9.78E-03	2.05E-05	6.18E-10	3.75E-08	
GO:0016740	Transferase activity	MF			7.45E-08		
GO:0097367	Carbohydrate derivative binding	MF			8.55E-06	1.50E-05	
GO:0032553	Ribonucleotide binding	MF			1.35E-05		
GO:0035639	Purine ribonucleoside triphosphate binding	MF	4.47E-02				
GO:0005524	ATP binding	MF	4.47E-02		1.67E-05	7.63E-06	
GO:0001883	Purine nucleoside binding	MF	4.47E-02	8.32E-05		1.63E-05	
GO:0032549	Ribonucleoside binding	MF	4.47E-02	8.32E-05		1.63E-05	
GO:0032550	Purine ribonucleoside binding	MF	4.47E-02	8.32E-05		1.63E-05	
GO:0001882	Nucleoside binding	MF	4.47E-02	8.32E-05			
GO:0032555	Purine ribonucleotide binding	MF	4.47E-02	8.32E-05			
GO:0032559	Adenyl ribonucleotide binding	MF	4.47E-02	4.72E-05	8.86E-06	6.07E-06	
GO:0030554	Adenyl nucleotide binding	MF		6.26E-05	1.08E-05	4.95E-06	
GO:0017076	Purine nucleotide binding	MF	4.62E-02	1.94E-04		1.63E-05	
GO:0001071	Nucleic acid binding transcription factor activity	MF					1.60E-02
GO:0003700	Transcription factor activity, sequence-specific DNA binding	MF					1.60E-02

* *BP, biological process; CC, cellular component; MF, molecular function*.

† *No GO term in the up-regulated DEGs was enriched in HSVd-h-infected cucumber plants at 2 dpi*.

Table 2 contains a comparable list of enriched GO terms for the down-regulated DEGs. While no GO terms were enriched for down-regulated DEGs in HSVd-h-infected plants at 2 and 14 dpi, 20 GO terms related to microtubule-based movement, microtubule binding, and microtubule motor activity were enriched at 28 dpi. Conversely, no GO terms were enriched for down-regulated DEGs in HSVd-g54-infected plants at 2 dpi, but 11 GO terms related to photosystem, thylakoid, photosynthetic membrane, and the photosystem I reaction center were enriched at 14 dpi.

**Table 2:** All enriched GO terms for the down-regulated DEGs in HSVd-infected cucumber plants.

**GO accession**	**Description**	Term type^*^	**Corrected *P*-value^†^**		
			**HSVd-h**	**HSVd-g54**	
			**28 dpi**	**14 dpi**	**28 dpi**
GO:0007018	Microtubule-based movement	BP	6.51E-14		
GO:0006928	Movement of cell or subcellular component	BP	3.42E-09		
GO:0007017	Microtubule-based process	BP	1.78E-07		
GO:0006260	DNA replication	BP	7.00E-03		
GO:0006269	DNA replication, synthesis of RNA primer	BP	3.61E-02		
GO:0015979	Photosynthesis	BP		1.09E-11	
GO:0055114	Oxidation-reduction process	BP			2.43E-02
GO:0048046	Apoplast	CC	8.01E-03		
GO:0005618	Cell wall	CC	3.66E-02		
GO:0009521	Photosystem	CC		3.16E-12	
GO:0009579	Thylakoid	CC		3.42E-12	
GO:0044436	Thylakoid part	CC		3.42E-12	
GO:0034357	Photosynthetic membrane	CC		4.18E-12	
GO:0009522	Photosystem I	CC		3.63E-07	
GO:0009523	Photosystem II	CC		2.00E-05	
GO:0098796	Membrane protein complex	CC		8.63E-04	
GO:0009538	Photosystem I reaction center	CC		9.51E-04	
GO:0042651	Thylakoid membrane	CC		1.99E-03	
GO:0009654	Photosystem II oxygen evolving complex	CC		3.10E-02	
GO:0008017	Microtubule binding	MF	9.05E-15		
GO:0003777	Microtubule motor activity	MF	9.05E-15		
GO:0015631	Tubulin binding	MF	1.67E-14		
GO:0003774	Motor activity	MF	9.29E-12		
GO:0032403	Protein complex binding	MF	3.42E-09		
GO:0008092	Cytoskeletal protein binding	MF	2.61E-07		
GO:0044877	Macromolecular complex binding	MF	2.14E-06		
GO:0017111	Nucleoside-triphosphatase activity	MF	2.07E-04		
GO:0016462	Pyrophosphatase activity	MF	7.78E-04		
GO:0016818	Hydrolase activity, acting on acid anhydrides, in phosphorus-containing anhydrides	MF	2.01E-03		
GO:0016817	Hydrolase activity, acting on acid anhydrides	MF	7.00E-03		
GO:0005198	Structural molecule activity	MF	7.00E-03		
GO:0016762	Xyloglucan:xyloglucosyl transferase activity	MF	8.01E-03		
GO:0016705	Oxidoreductase activity, acting on paired donors, with incorporation or reduction of molecular oxygen	MF			4.01E-05
GO:0020037	Heme binding	MF			4.01E-05
GO:0046906	Tetrapyrrole binding	MF			4.01E-05
GO:0005506	Iron ion binding	MF			2.77E-04
GO:0003824	Catalytic activity	MF			2.43E-02

* *BP, biological process; CC, cellular component; MF, molecular function*.

† *No GO term in the down-regulated DEGs was enriched in HSVd-h-infected cucumber plants at 2 and 14 dpi and HSVd-g54 at 2 dpi*.

### KEGG enrichment analysis

Many different gene-encoded products function *in vivo* as part of one or more biochemical pathways. To determine in which major metabolic and signal transduction pathways might be disrupted by HSVd infection, we performed a KEGG enrichment analysis (Table 3, Table S6).

**Table 3:** KEGG pathway enrichment of DEGs from HSVd-infected cucumber plants.

**Pathway**	**ID**	**HSVd-h**						**HSVd-g54**					
		**2 dpi**		**14 dpi**		**28 dpi**		**2 dpi**		**14 dpi**		**28 dpi**	
		No.^*^	**q^†^**	**No**.	**q**	**No**.	**q**	**No**.	**q**	**No**.	**q**	**No**.	**q**
**UP-REGULATED ENRICHMENT KEGG PATHWAYS (CORRECTED *P*-VALUE < 0.05)**													
Pentose phosphate pathway	csv00030	2	3.03E-02										
Plant hormone signal transduction	csv04075					44	1.03E-13	20	3.09E-02	13	2.74E-03	13	1.81E-06
Plant-pathogen interaction	csv04626					21	3.07E-06	28	1.47E-11			5	3.87E-02
Endocytosis	csv04144							12	2.08E-02				
**DOWN-REGULATED ENRICHMENT KEGG PATHWAYS (CORRECTED *P*-VALUE < 0.05)**													
Photosynthesis—antenna proteins	csv00196	8	4.99E-09					14	8.00E-15	10	1.46E-09		
Glutathione metabolism	csv00480	7	1.44E-04										
Protein processing in endoplasmic reticulum	csv04141	9	8.58E-04										
Plant-pathogen interaction	csv04626			2	1.40E-02								
DNA replication	csv03030					16	3.47E-04						
Porphyrin and chlorophyll metabolism	csv00860					11	1.96E-02						
Ribosome	csv03010					42	4.05E-02						
Plant hormone signal transduction	csv04075							15	2.27E-03				
Photosynthesis	csv00195									21	1.85E-16		
Flavonoid biosynthesis	csv00941											4	3.98E-04
Biosynthesis of secondary metabolites	csv01110											17	7.16E-03
Phenylpropanoid biosynthesis	csv00940											6	1.35E-02
Phenylalanine metabolism	csv00360											5	1.86E-02

* No., Input number;

† *q, Corrected P-value (< 0.05)*.

Four KEGG pathways were enriched in up-regulated DEGs: the pentose phosphate pathway, plant hormone signal transduction, plant-pathogen interaction, and endocytosis. Plant hormone signal transduction and plant-pathogen interaction were the main pathways affected, because they were enriched in plants infected with HSVd-g54 at 2 and 28 dpi as well as HSVd-h at 28 dpi. Hormone signal transduction was enriched in plants infected with HSVd-g54 at 14 dpi, while the pentose phosphate pathway and endocytosis were enriched at 2 dpi in plants infected with HSVd-h and HSVd-g54 (Table 3).

Thirteen KEGG pathways were enriched in down-regulated DEGs. Photosynthesis-antenna proteins were enriched in plants infected with HSVd-h at 2 dpi and HSVd-g54 at 2 and 14 dpi. Other down-regulated pathways were enriched only at a single time point (Table 3).

### HSVd infection induces expression of many basal defense response-related genes

Plants have evolved a variety of defense responses to prevent or limit disease. Many of these responses are activated locally at the site of infection and then spread systemically when a plant is under pathogen attack (Whitham et al., 2006; Pallas and García, 2011; Allie et al., 2014). This initial response is usually termed basal or broad spectrum immunity and may be sufficient to combat a viral pathogen.

Activation of the defense response results from several possible signaling pathways, including reactive oxygen species (ROS), signaling molecules, and pathogenesis-related proteins (PR proteins), leading to biochemical and morphological alterations such as cell wall reinforcement and transmembrane reconfiguration in the host (Fagard et al., 2007; Blomster et al., 2011). KEGG pathway analysis revealed that many genes involved in plant basal defense responses were expressed at higher levels in HSVd-inoculated cucumber plants (Table S7). Compared with mock-inoculated plants, genes encoding cyclic nucleotide gated channels (CNGCs), calcium-dependent protein kinase (CDPK), respiratory burst oxidase (Rboh), calcium-binding protein CML (CaMCML), WRKY transcription factor 33 (WRKY33), LRR receptor-like serine/threonine-protein kinase FLS2 (FLS2), mitogen-activated protein kinase kinase kinase 1 (MEKK1), brassinosteroid insensitive 1-associated receptor kinase 1 (BAK1/BKK1), the basic form of PR1, and mitogen-activated protein kinase kinase 4/5 (MKK4/5) were all up-regulated at different time points (Table S7). The number of up-regulated DEGs involved in basal defense responses increased over the time course of the experiment for the HSVd-h vs. mock control, while the number of basal defense response-related genes declined in the HSVd-g54 vs. mock control. It should be noted that the *PR1* gene was expressed at extremely low levels in mock-inoculated cucumber plants but was up-regulated in plants infected with either HSVd-h or HSVd-g54 at 14 and 28 dpi (Table S7).

### HSVd infection represses expression of photosynthesis-related genes

To better understand the molecular effects of HSVd infection in cucumber, we annotated the DEGs identified in HSVd-h- and HSVd-g54-inoculated tissue harvested at all time points using the GO and KEGG terms. For HSVd-g54-infected systemic leaves harvested at 14 dpi, almost all of the down-regulated GO terms were in the cellular component category. These genes were involved in chloroplast functions including photosystem I and II (PS I, PS II), thylakoids and photosynthesis (Table 2). At 2 dpi the most strongly down-regulated KEGG terms in the HSVd-h vs. mock and HSVd-g54 vs. mock controls were photosynthesis-antenna proteins (Table 3). At 14 dpi two of the down-regulated KEGG terms in the HSVd-g54 vs. mock control were photosynthesis and photosynthesis-antenna proteins. At 2 dpi, only the genes encoding the light-harvesting chlorophyll (LHC) complex proteins Lhca2, Lhca4, Lhcb1, Lhcb2, and Lhcb3 were down-regulated in HSVd-h-infected cucumber plants; in the HSVd-g54-infected plants, in contrast, genes encoding Lhca1, Lhca2, Lhca3, Lhca4, Lhcb1, Lhcb2, Lhcb3, Lhcb5, and Lhcb6 were down-regulated. At 14 dpi, genes encoding photosystem I and II proteins PsbO, PsbP, PsbQ, PsbW, PsbY, PsaD, PsaE, PsaF, PsaG, PsaH, PsaK, PsaL, PsaN, and PsaO, as well as genes encoding photosynthetic electron transport proteins PetE and PetH and the F-type ATPase subunits b and γ were down-regulated in HSVd-g54-infected plants.

**Figure 5:**
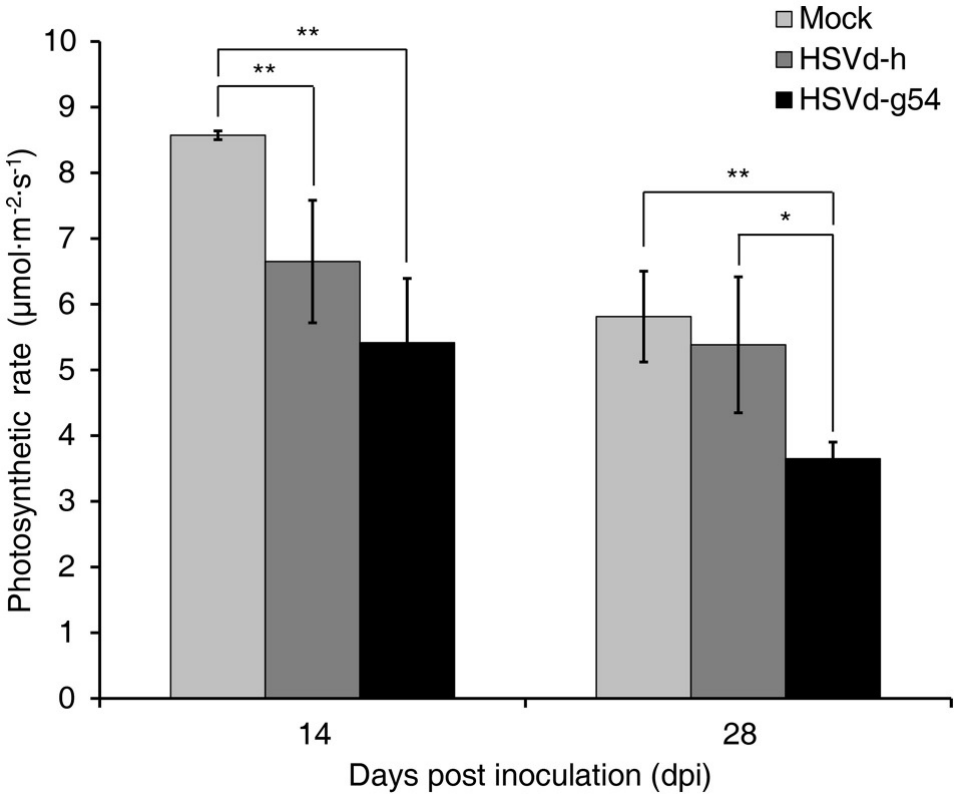
Effect of HSVd infection on the rate of photosynthesis in cucumber. Experiments were performed on six cucumber plants, and the data are expressed as the mean ± standard deviation. Error bars indicate the standard deviations of six replicates. Single or double asterisks indicate statistically significant differences compared with the control at *p* < 0.05 or 0.01, respectively, in Student's *t-*test.

To determine whether or not these changes in expression levels affected photosynthesis, the photosynthetic rates of cucumber plants inoculated with HSVd-h and HSVd-g54 were measured with a LI-6400 Portable Photosynthesis System at 14 and 28 dpi and then compared to those of mock-inoculated plants normalized according to the total leaf area. The results showed that the photosynthetic rate in HSVd-h- and HSVd-g54-infected plants declined dramatically as compared to the control plants at 14 dpi. Interestingly, differences between plants infected with HSVd-h and HSVd-g54 were not statistically significant (Figure 5). At 28 dpi, photosynthetic rates in cucumber plants infected with HSVd-g54 decreased sharply compared to those in HSVd-h-infected or mock-inoculated plants: no significant difference was observed between the HSVd-h and mock-inoculated plants. These results indicated that infection with HSVd inhibits photosynthesis in cucumber plants.

### HSVd infection induces expression of salicylic acid-related genes

**Figure 6:**
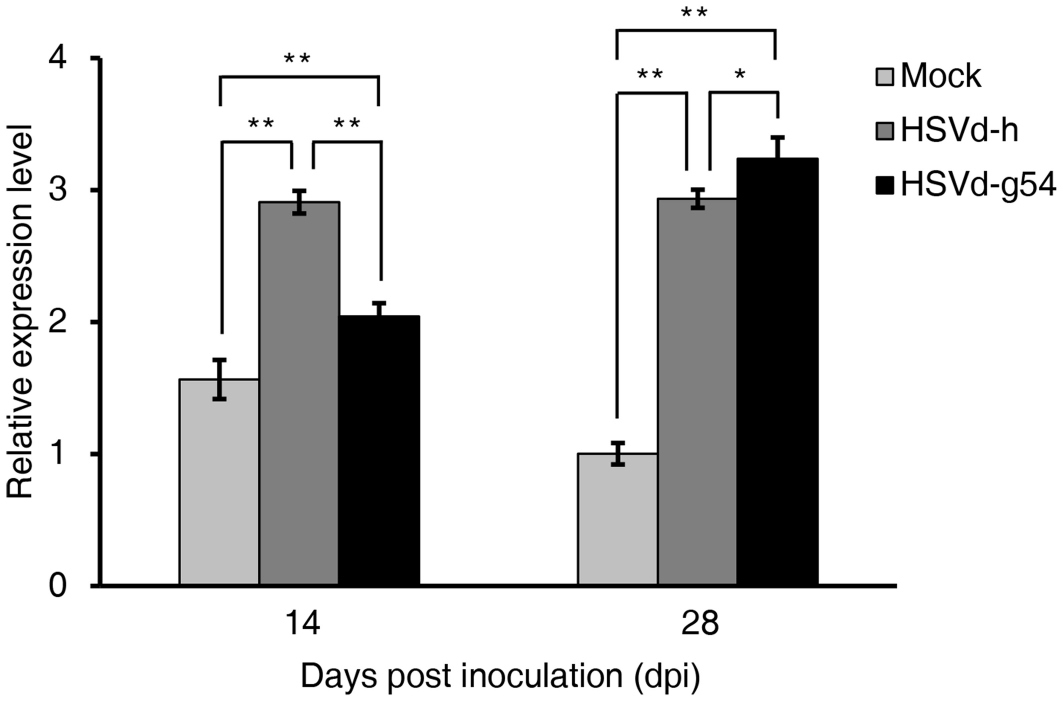
Effect of HSVd infection on *PR1* gene expression. Experiments were performed in triplicate and the data are expressed as the mean ± standard deviation. Error bars indicate the standard deviations of three biological replicates of RT-qPCR analysis. Single or double asterisks indicate statistically significant differences compared with the control at *P* < 0.05 or 0.01, respectively, in Student's *t-*test. *EF1*α was used as the reference gene to normalize the gene expression.

Phytohormones play critical roles in modulating plant growth and development, and infection by many viruses and viroids can affect several kinds of phytohormone signaling pathways as well as their biosynthesis and catabolism, resulting in the appearance of disease symptoms in the infected plants (Whenham et al., 1986; Owens et al., 2012). In this study, we also used KEGG enrichment analysis to identify DEGs related to phytohormone signal transduction. In cucumber plants infected with HSVd-h, phytohormone signal transduction was the top KEGG term at both 14 and 28 dpi. Four DEGs were involved in the IAA, ET, JA, and SA signal transduction pathways at 14 dpi; at 28 dpi the number of DEGs increased to 57, including most of the major phytohormone signaling pathways (Table S8). Compared with mock-inoculated plants, cucumber plants infected with HSVd-g54 had 35, 18, and 13 DEGs related to phytohormone signal transduction at 2, 14, and 28 dpi, respectively (Table S8). Especially noteworthy was the up-regulation of genes encoding the main components of the SA signaling pathway in HSVd-infected plants. *NPR1* (*Non-expressor of pathogenesis-related genes 1*) was up-regulated in HSVd-h-infected plants at 28 dpi and HSVd-g54 infected plants at 14 dpi. *PR1*, which is also known as a marker gene for SA (Divi et al., 2010; Ford et al., 2010), was up-regulated in both HSVd-h- and HSVd-g54-infected plants at 14 and 28 dpi (Figure 6, Table S3). This suggested that HSVd-infected plants may contain higher concentrations of SA than healthy cucumber plants.

### HSVd infection induces expression of *CsRDR1* genes

RNA silencing not only plays a critical role in plant antiviral response but is also directly involved in viral disease induction in plants (Navarro et al., 2012; Wang et al., 2012). We therefore screened the cucumber DEGs associated with HSVd infection for the presence of RNA silencing-related genes encoding Dicer-Like enzymes (DCL), Argonaute proteins (AGO), and RDR. *DCL1* and *DCL4* were slightly up-regulated, and *AGO7, AGO10*, and *AGO16* were down-regulated more than two-fold at 28 dpi in HSVd-h-infected plants. Notably, the expression of genes encoding components of *RDR1* was changed dramatically in both HSVd-h- and HSVd-g54-infected plants at various time points (Table 4). Cucumber *RDR1* consists of *CsRDR1a, CsRDR1b, CsRDR1c1*, and *CsRDR1c2* (Leibman et al., 2017). *CsRDR1a* expression was slightly stimulated in HSVd-h-infected cucumber plants at 28 dpi, and expression of *CsRDR1b* increased slightly in HSVd-h-infected plants at 28 dpi and HSVd-g54-infected plants at 14 and 28 dpi (Table 4). In contrast to *CsRDR1a* and *CsRDR1b*, expression of *CsRDR1c1* and *CsRDR1c2* increased dramatically in HSVd-infected cucumber plants. At 28 dpi, expression of *CsRDR1c1* was highly up-regulated in both HSVd-h- and HSVd-g54-infected plants; expression of *CsRDR1c2* was significantly induced in both HSVd-h- and HSVd-g54-infected cucumber plants at both 14 and 28 dpi (Table 4). These results obtained using RNA-seq were validated by RT-qPCR, and data summarized in Table 4 show that both methods detected similar changes in *CsRDR1* gene expression.

**Table 4:** Relative expression of cucumber *RDR1* genes in HSVd infected plants.

**Pathogen**	**Time (dpi)**	**Gene name**	**Gene ID**	**log**_**2**_**FoldChange**	
				**RNA-seq**	**RT-qPCR**
HSVd-h	14	*RDR1c2*	LOC101213473	4.03	4.20
	28	*RDR1c2*	LOC101213473	4.79	15.86
		*RDR1c1*	LOC101205704	3.44	51.51
		*RDR1b*	LOC101206946	1.13	3.55
		*RDR1a*	LOC101206705	0.65	1.67
HSVd-g54	14	*RDR1c2*	LOC101213473	5.17	20.60
		*RDR1b*	LOC101206946	1.46	3.35
	28	*RDR1c2*	LOC101213473	4.58	19.06
		*RDR1c1*	LOC101205704	3.45	33.87
		*RDR1b*	LOC101206946	1.25	4.03

## Discussion

In this study we examined the changes in the cucumber transcriptome that occur following infection by mild (HSVd-h) and severe (HSVd-g54) variants of HSVd. An earlier study compared differences in the tomato transcriptome caused by infection with mild and severe strains of PSTVd using subtracted cDNA libraries (Itaya et al., 2002). Two other studies focused on the changes in host gene expression following either infection by citrus exocortis viroid (CEVd) (Rizza et al., 2012) or PSTVd infection in two different tomato cultivars (Owens et al., 2012). Each of these studies used macroarray or microarray analysis to assess the host response to a single viroid variant at a single time point. Here, we have used RNA-seq analysis to compare changes in cucumber gene expression associated with infection by both a mild and severe variants of HSVd during the early (2 dpi), middle (pre-symptomatic, 14 dpi), and late (post-symptomatic, 28 dpi) stages of infection. The results described highlight the transcriptomic changes caused by HSVd infection, changes affecting genes involved in basal defense responses, photosynthesis, and SA regulation as well as in *CsRDR1*.

### Viroid infection triggers basal defense responses

Plants have evolved an innate immune system that recognizes invading microbes and initiates an effective defense response against pathogen attack (Wu et al., 2014). These are known as basal defense responses. When pathogen invasion occurs, plants translate perception of the pathogen into signal cascades involving CNGCs that raise Ca^2+^ concentrations in the cytosol and activate CaMs and/or CaM-like proteins (CMLs) (Ali et al., 2007). CDPKs are another major type of Ca^2+^ sensor in plants, and they can be activated by Ca^2+^. CDPK cascades are initiated downstream of the activated receptor complex and further mediate defense gene expression (Wu et al., 2014).

*Arabidopsis* CDPK1, 2, 4, and 11 strongly phosphorylate both RbohD and RbohF *in vitro* and mediate AvrRpm1-induced ROS production (Gao et al., 2013). Expression of CNGCs, CaMs/CMLs, CDPKs, and Rboh was changed in cucumber plants infected with HSVd-h at 28 dpi and HSVd-g54 at 2 and 14 dpi (Table S7). Expression of FLS2, an LRR receptor-like kinase usually involved in perception of the bacterial elicitor flagellin, was also activated by HSVd infection, and it can associate with BAK1 to form the FLS2/BAK1 complex (Gómez-Gómez and Boller, 2000; Wu et al., 2014). The signal can then be passed from FLS2 to MEKK1, and accumulating evidence suggests that in *Arabidopsis* perception activates two branches of the MAPK cascade: MEKK1/MEKKs-MKK4/5-MPK3/6 and MEKK1-MKK1/2-MPK4 (Tena et al., 2011; Wu et al., 2014).

Pathogen infection often leads to transcriptional changes in the plant host. A very mild response usually does not result in large-scale changes in gene expression patterns. However, a hypersensitive response (HR) is accompanied by massive increases in the levels of a large number of different proteins. Among these induced proteins are members of a group of PR proteins (Linthorst and Van Loon, 1991). When infected by apple stem grooving virus (ASGV), apple genes encoding PR proteins PR1 and PR1a were up-regulated (Chen et al., 2014); similarly, *PR1* expression in tomato was also induced by infection with tomato spotted wilt virus (TSWV) (López-Gresa et al., 2016) and CEVd (Conejero et al., 1990; López-Gresa et al., 2016). The up-regulation of cucumber *PR1* gene expression, which we observed following either HSVd-h or HSVd-g54 infection (Table S7), is consistent with the results of these previous studies.

### Viroid infection represses plant photosynthesis

The photosynthetic rate in HSVd-infected cucumber plants was reduced (Figure 5). Many genes associated with photosynthesis were found to be down-regulated in cucumber plants infected with HSVd-h or HSVd-g54 compared to mock-inoculated plants. The organic compounds and energy required for plant growth comes from photosynthesis, and limiting photosynthesis may repress growth and development, which may be one of the causes of dwarfing observed in cucumber plants inoculated with HSVd, especially those inoculated with the severe mutant HSVd-g54.

Viroid infection could modify the structure or synthesis of chloroplast, which affect the efficiency of photosynthesis. There are many studies showing that virus infection represses photosynthesis in the host (Li et al., 2016). Virus infection can impair photosynthesis by decreasing carboxylation and mesophyll conductance (Sampol et al., 2003), reducing carboxylative efficiency (Pérez-Clemente et al., 2015), modifying the structure of chloroplasts, or reducing the number of active reaction centers (Prod'homme et al., 2001; Zechmann et al., 2003; Bhattacharyya et al., 2015; Otulak et al., 2015). Previous studies also reported that the chloroplast structure of viroid-infected plant tissues was modified, particularly thylakoid membrane abnormalities and paucity of grana in many viroid-host combinations (Hari, 1980; da Graça and Martin, 1981; Kojima et al., 1983; Momma and Takahashi, 1983). The infection of peach latent mosaic viroid (PLMVd) with a specific structural motif (12- to 13-nucleotide insertion) inhibited chloroplast development (Rodio et al., 2007). Microarray analysis indicated that viroid infection triggered changes in chloroplast, and its biogenesis-related genes were down-regulated (Owens et al., 2012; Rizza et al., 2012). In this study, we annotated the down-regulated genes associated with photosynthesis GO and KEGG terms. Proteins encoded by these genes included photosynthesis-antenna proteins (components of LHC in PS I and PS II), photosynthetic electron transport proteins PetE and PetH, and the F-type ATPase subunits b and γ. Thus, HSVd infection down-regulates the expression of several genes involved in photosynthesis, genes that could explain the decrease in photosynthetic rate observed in the infected plants.

### Role of SA in the interaction between viroid and plants

HSVd infection induces the expression of the SA marker gene *PR1* in cucumber plants, and SA may be involved in the interaction between viroid and plants. During infection by viruses and viroids, disruption of normal host developmental physiology is often associated with alterations in phytohormone production, accumulation, and signaling (Whenham et al., 1986; Zhu et al., 2005; Hammond and Zhao, 2009; Owens et al., 2012; Rodriguez et al., 2014; Collum and Culver, 2016).

In our study, most of the major plant hormone signal transduction pathways were found to be affected at different times after infection by the two HSVd variants tested. And this indicated HSVd infection changed many plant hormone signal transduction pathways simultaneously, and the relationship between viroid, phytohormone, and host plant is complex. However, some regulation by plant hormones was still present. For example, genes encoding some components of the SA signal transduction pathway were up-regulated by both HSVd-h and HSVd-g54 at 14 and 28 dpi; this was especially true for *PR1*, a known SA marker gene (Divi et al., 2010; Ford et al., 2010), that is expressed at very low levels in healthy cucumber plants. *PR1* was up-regulated significantly in HSVd-infected plants at 14 and 28 dpi (Figure 6, Table S3), indicating that SA may accumulate to higher levels in HSVd-infected cucumber plants as a result of viroid infection. Our results are consistent with those of previous studies. For example, Zheng and colleagues found that genes involved in SA biogenesis and responses, auxin responses, and ethylene biogenesis and responses were induced in PSTVd-infected tomato plants (Zheng et al., 2017).

SA could enhance the resistance of plants to viroid or virus infection. SA activated plant defenses to CEVd and tomato mosaic virus (ToMV) in tomato (Bellés et al., 1999). Plum pox virus (PPV) was able to move to upper non-inoculated leaves of tobacco plants expressing bacterial salicylate hydroxylase (NahG) that degrades SA. Accumulation of virus-derived small RNAs was reduced in the NahG transgenic plants, suggesting that SA might act as an enhancer of the RNA silencing antiviral defense in tobacco (Alamillo et al., 2006). SA treatment induced resistance to CEVd and ToMV in tomato and *Gynura auriantiaca*, and SA accumulation occured in plants infected by CEVd and ToMV. The RNA silencing-related genes *ToDCL1, ToDCL2, ToRDR1*, and *ToRDR2* were significantly induced by both SA and CEVd in tomato, whereas *ToDCL4* and *ToRDR6* were only induced by CEVd (Campos et al., 2014). Therefore, both RNA-silencing and SA-mediated defense mechanism could act together to limit viroid and virus infection. Comparing with the corresponding parental plants, the lack of SA accumulation in the CEVd- or TSWV-infected NahG transgenic tomato plants led to early symptoms, thus showing that SA is an important component of basal resistance of tomato plants to CEVd and TSWV (López-Gresa et al., 2016). Silencing of SA biosynthetic and signaling genes in *Nicotiana benthamiana* plants increased susceptibility to tobacco mosaic virus (TMV), indicating that SA was required for systemic resistance response against this virus (Zhu et al., 2014). Therefore, SA may activate plant defenses to viroid and virus infection.

In addition, in the GO analysis of DEGs in HSVd-infected vs. mock-inoculated cucumber plants, we analyzed the top 20 up-regulated GO terms at all three time points and found that about 50% of the top 20 GO terms were related to protein kinase activity, protein phosphorylation, phosphotransferase activity, and transferase activity (transferring phosphorus-containing groups) (Table 1). This observation is consistent with previous reports indicating that PKV (protein kinase-viroid induced) is activated in tomato plants infected with PSTVd (Hammond and Zhao, 2000, 2009). Furthermore, PKV may regulate plant development by functioning in critical signaling pathways involved in gibberellic acid metabolism (Hammond and Zhao, 2009).

### Response of *CsRDR1* gene to viroid infection

*RDR1* plays an important role in the host response to biotic and abiotic stresses. It is induced by both virus infection (Liu et al., 2009; Leibman et al., 2017) and SA treatment (Xie et al., 2001; Wassenegger and Krczal, 2006; Liu et al., 2009). Thus, it was not unexpected to find that HSVd infection also induces *RDR1* expression. *CsRDR1a* and *CsRDR1b* expression was only weakly induced at one or two time points; *CsRDR1c1*, in contrast, was strongly induced by both HSVd-h and HSVd-g54 at 28 dpi, and *CsRDR1c2* was highly induced by both variants at 14 and 28 dpi (Table 4).

We validated the results of RNA-seq analysis of *CsRDR1* gene expression by RT-qPCR. Even though the values obtained by the two methods were different, the data revealed similar trends in the level of *CsRDR1* gene expression (Table 4). Several factors (e.g., gene length, GC content, the method of constructing the library for RNA-seq, and the algorithm used to measure gene expression) can affect results obtained by RNA-seq (Hansen et al., 2010; Zheng et al., 2011; SEQC/MAQC-III Consortium, 2014; Robert and Watson, 2015; Love et al., 2016), and low expression levels of *CsRDR1c1* and *CsRDR1c2* in mock-inoculated cucumber plants (Leibman et al., 2017) may cause the different results of the two methods. The induction of *RDR1* gene expression in response to HSVd infection observed in our experiments is consistent with that previously found in PSTVd-infected tomato (Schiebel et al., 1998), suggesting that *CsRDR1* may play an important role in viroid-host interaction.

Early studies presenting evidence for RNA silencing as a mediator of host-viroid interaction have been reviewed by Ding (2009), and RDR1 is a major component of RNA silencing pathways (Ahlquist, 2002) contributing to basal resistance to several viruses through its role in the biogenesis of virus-derived secondary siRNAs (Diaz-Pendon et al., 2007; Qi et al., 2009; Garcia-Ruiz et al., 2010; Wang et al., 2010b). *RDR1* is also responsible for production of a distinct class of virus-activated siRNAs (vasiRNAs), which direct widespread silencing of host genes and confer broad-spectrum antiviral activity in *Arabidopsis* (Cao et al., 2014). Taken together, these studies indicate that *RDR1* plays key role in antiviral resistance by silencing both viral RNAs and host immunity-related genes.

In *Arabidopsis* RNA silencing-mediated viral immunity requires *RDR1* and *RDR6* to amplify virus-derived siRNAs (Wang et al., 2010b). Previous studies have reported *RDR1* orthologs to be virus-, viroid-, or SA-inducible in different plants (Schiebel et al., 1998; Xie et al., 2001; Yang et al., 2004; Alamillo et al., 2006; He et al., 2010; Leibman et al., 2017); furthermore, a correlation between SA-mediated and RNA-silencing defenses has been suggested (Xie et al., 2001). In this study, HSVd infection was shown to induce some genes involved in the SA signaling pathway as well as *CsRDR1*. To determine whether or not the induction of *CsRDR1* by HSVd infection is directly mediated by SA will require additional research. Besides *RDR1*, some studies have reported that *RDR2* (Campos et al., 2014) and *RDR6* (Gómez et al., 2008; Di Serio et al., 2010) are also involved in viroid-host interactions. Our studies failed to find any evidence for changes in *RDR2* or *RDR6* transcription during HSVd infection in cucumber, but these differences may be due to the use of different viroids and host plant species.

In conclusion, we have investigated the global mRNA transcriptional profiles for cucumber plants infected with either a mild (HSVd-h) or a severe (HSVd-g54) variant of HSVd and compared those profiles to the profile in uninfected plants. Identification of DEGs in the present study shows that HSVd-g54 induces an earlier and stronger response than HSVd-h. HSVd infection triggers basal defense responses, depresses photosynthesis, disrupts phytohormone homeostasis, may induce the accumulation and signal transduction of SA, and induces the expression of *CsRDR1* genes, especially *CsRDR1c1* and *CsRDR1c2*, in cucumber plants. Our findings will accelerate research on the interaction between plants and viroids and contribute to a better understanding of the mechanisms that determine viroid pathogenicity.

## Data deposition

The raw RNA-seq data were deposited in the NCBI Sequence Read Archive (SRA), and the Accession number is SRR6122525.

## Author contributions

CX: conducted lab experiments, analyzed the data, and drafted the manuscript; ZZ, TS, and SL: participated in design of the experiments, analyzed the data, and revised the manuscript; WH, ZF, HX, and ML: revised the manuscript. All authors read and approved the final manuscript.

### Conflict of interest statement

The authors declare that the research was conducted in the absence of any commercial or financial relationships that could be construed as a potential conflict of interest. The handling Editor declared a past co-authorship with one of the authors SL and TS.

## Supplementary material

Figure S1 Original images of the Northern-blot and the agarose gel before transfer. **(A)** Original image of the Northern-blot. **(B)** Original image of the agarose gel before transfer. M, mock; h, cucumber infected with HSVd-h; g54, cucumber infected with HSVd-g54; PC, positive control (plasmid containing HSVd cDNA dimer).

Figure S2 Correlation between individual samples (mock, HSVd-h, and HSVd-g54 infection; 2, 14, and 28 dpi) and the comparison of gene expression levels among samples. **(A)** Pearson correlation between samples. An *R*^2^ value close to 1 indicates a high degree of correlation between samples. **(B)** FPKM distribution. FPKM: expected number of Fragments Per Kilobase of transcript sequence per Million base pairs sequenced. M, mock; h, cucumber with HSVd-h infection; g54, cucumber with HSVd-g54 infection.

Table S1 Oligonucleotide primers used for RT-qPCR analyses.

Table S2 Statistics of RNA sequencing reads, data quality, Q-scores, and clean reads mapping to the reference genome for nine independent samples isolated from HSVd-infected cucumber plants.

Table S3 Complete list of DEGs detected in this study.

Table S4 Validation of RNA-seq results by RT-qPCR.

Table S5 List of GO terms describing DEGs identified in this study.

Table S6 List of KEGG pathways containing DEGs identified in this study.

Table S7 Effect of HSVd infection on the expression of cucumber genes involved in the basal defense response.

Table S8 KEGG pathway analysis of plant hormone signal transduction in HSVd-infected cucumber. Results for HSVd-h vs. mock, HSVd-g54 vs. mock, and HSVd-g54 vs. HSVd-h comparisons are shown.
