# Supplementary figures and images for: Global Transcriptomic Changes Induced by Infection of Cucumber (Cucumis sativus L.) with Mild and Severe Variants of Hop Stunt Viroid
Source: Front Microbiol. 2017 Dec 12;8:2427. doi: 10.3389/fmicb.2017.02427 (PMC5733102; doi:10.3389/fmicb.2017.02427)

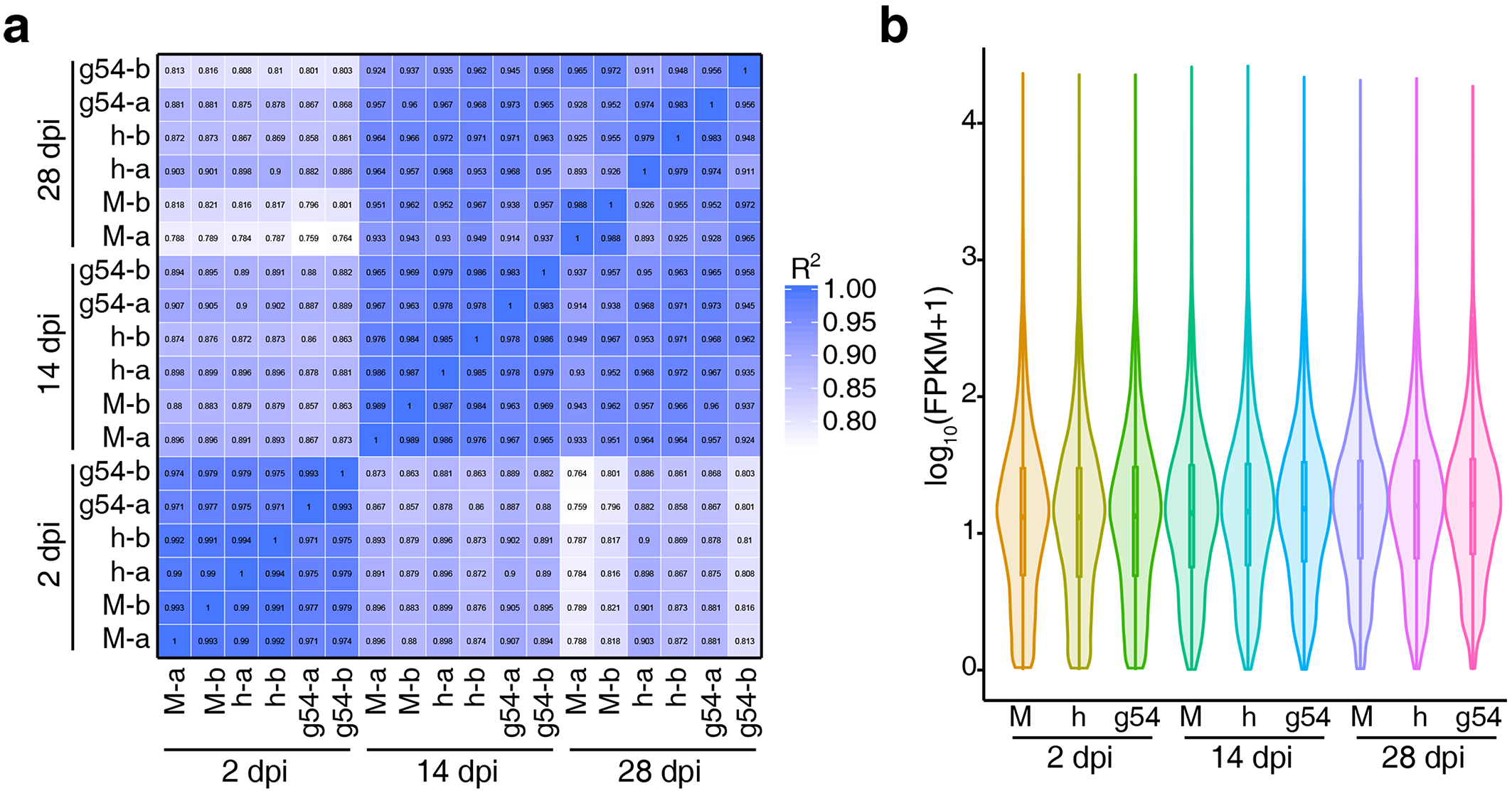

Supplement: Figure S1 — Original images of the Northern-blot and the agarose gel before transfer. (A) Original image of the Northern-blot. (B) Original image of the agarose gel before transfer. M, mock; h, cucumber infected with HSVd-h; g54, cucumber infected with HSVd-g54; PC, positive control (plasmid containing HSVd cDNA dimer). [file Image1.TIF]

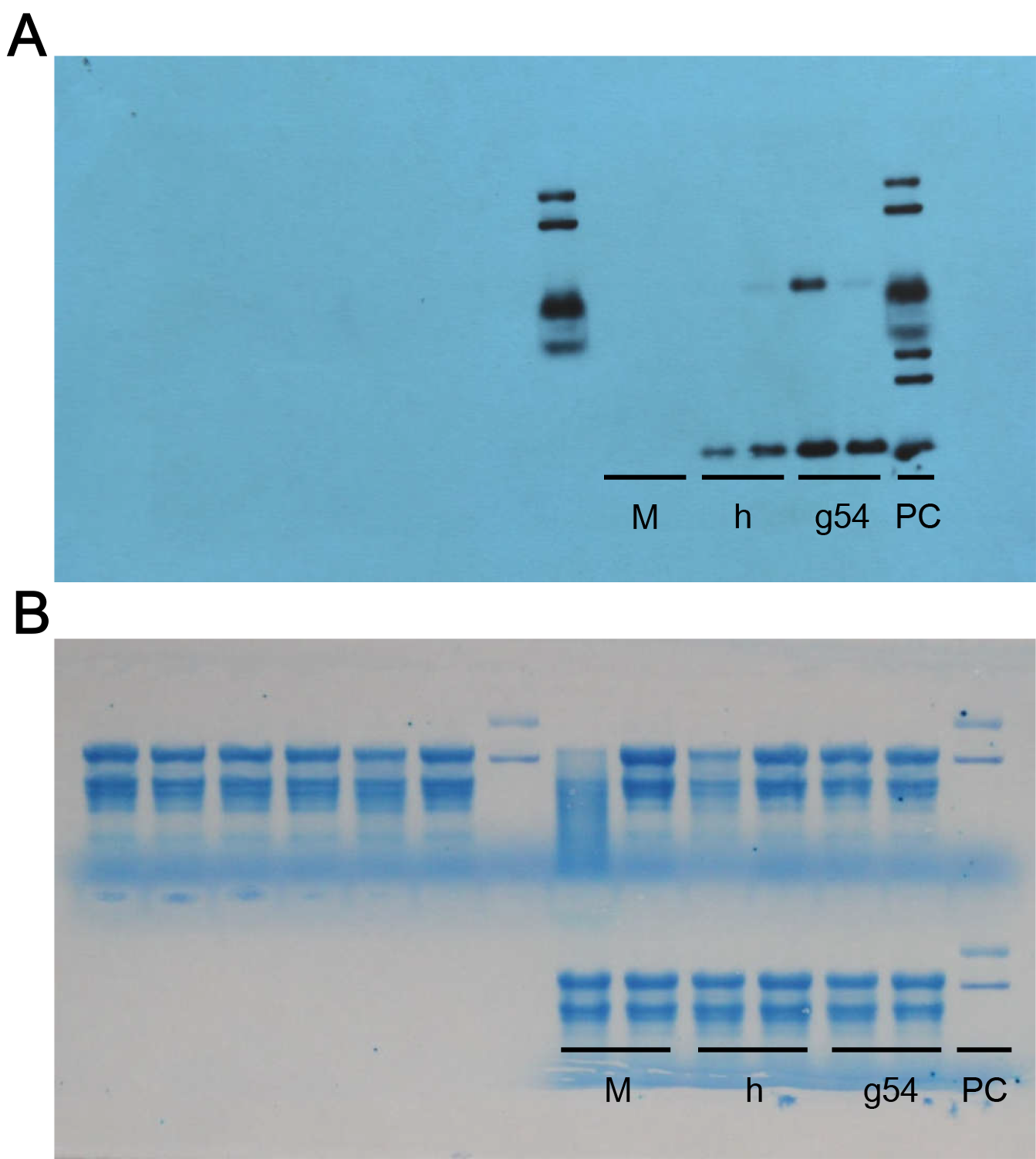

Supplement: Figure S2 — Correlation between individual samples (mock, HSVd-h, and HSVd-g54 infection; 2, 14, and 28 dpi) and the comparison of gene expression levels among samples. (A) Pearson correlation between samples. An R2 value close to 1 indicates a high degree of correlation between samples. (B) FPKM distribution. FPKM: expected number of Fragments Per Kilobase of transcript sequence per Million base pairs sequenced. M, mock; h, cucumber with HSVd-h infection; g54, cucumber with HSVd-g54 infection. [file Image2.TIF]
