# Supplementary material for: Global Transcriptomic Changes Induced by Infection of Cucumber (Cucumis sativus L.) with Mild and Severe Variants of Hop Stunt Viroid
Source: Front Microbiol. 2017 Dec 12;8:2427. doi: 10.3389/fmicb.2017.02427 (PMC5733102; doi:10.3389/fmicb.2017.02427)
Supplement: Table S1 — Oligonucleotide primers used for RT-qPCR analyses. [file Table1.docx]

Table S3 Oligonucleotide primers used for RT-qPCR analyses

| **Gene or viroid** | **Primer** | **Sequence (5'-3')** | **Reference** |
| --- | --- | --- | --- |
| HSVd | HSVd-F | GGAAGGTACTTACCTGAGAAAGG |  |
|  | HSVd-R | CCAGGAGAAGGTAAAGAAGAAGG |  |
| LOC101204004 | 004-F | CCAGATGGACAGAACAAGTGAA |  |
|  | 004-R | CTCGGATATGTTGCACCAAGTA |  |
| LOC101216568 | 568-F | TCCCAAGGTTGTTCCGTTTC |  |
|  | 568-R | GCCACCTCCTATTGAAGCATAC |  |
| LOC101204061 | 061-F | TCCCTCTCCGGTGAAGAATA |  |
|  | 061-R | GTTGCATTGATCCCACCAATC |  |
| LOC101205677 | 677-F | CAGCCCGTGTATAGACCTTTAG |  |
|  | 677-R | AGAGGCAGAGTGAAGTAGGA |  |
| LOC101212420 | 420-F | CCCACCATATCGCCAATTCT |  |
|  | 420-R | GTCGAACGGCCATCTGATAAT |  |
| LOC101213224 | 224-F | CCTTGTGGTTTGGGCTATCT |  |
|  | 224-R | CATACCCTCCTTCACCTACAATAC |  |
| LOC101208445 | 445-F | CCGCCGAGTCTGAAACTAAT |  |
|  | 445-R | GAATCAGAGAGACGGAGGTTATG |  |
| LOC101209949 | 949-F | GAGGTTGTCCACGAGTTTCA |  |
|  | 949-R | GAGGGCCATTCATCGACATAA |  |
| LOC101208068 | 068-F | GGGAGTTTGGAGTCGATGATTA |  |
|  | 068-R | GCTTCCAGAGTTACCTCCATAC |  |
| LOC101220230 | 230-F | AAGGCGAACTAAGCACTTCC |  |
|  | 230-R | GTTGGTGACACCCGACATTAT |  |
| *RDR1a* | RDR1a-F  RDR1a-R | CGTTCTCATGTTCTGCCGTA  TTCGACCAACCGGTAGAAAC | Leibman *et al*., 2017 |
| *RDR1b* | RDR1b-F  RDR1b-R | TAACAGCCGTGGATGTACCA  ATCGCTTCCAGAGCATTCAT | Leibman *et al.*, 2017 |
| *RDR1c1* | RDR1c1-F  RDR1c1-R | GCTACAAACCTGCACCAACA  CTCCAAGACCATCGTTCACC | Leibman *et al.*, 2017 |
| *RDR1c2* | RDR1c2-F  RDR1c2-R | CCTGCATTGGACAATCCTTTAC  TGGAGCATAGGTCACTTTGTAG |  |
| *PR1* | PR1-F  PR1-R | CCTCAAGACTTGGTCGGTGT  GTAAGGTCCGCCAGAGTTCA | Kuźniak et al., 2015 |
| *EF1α* | EF1α-F | ACTGTGCTGTCCTCATTATTG | Wan *et al*., 2010 |
|  | EF1α-R | AGGGTGAAAGCAAGAAGAGC |  |
