# Supplementary material for: Global Transcriptomic Changes Induced by Infection of Cucumber (Cucumis sativus L.) with Mild and Severe Variants of Hop Stunt Viroid
Source: Front Microbiol. 2017 Dec 12;8:2427. doi: 10.3389/fmicb.2017.02427 (PMC5733102; doi:10.3389/fmicb.2017.02427)
Supplement: Table S4 — Validation of RNA-seq results by RT-qPCR. [file Table4.DOCX]

Table S4 Validation of RNA-seq results by RT-qPCR

| Pathogen | Time (dpi) | Gene ID | log_2_FoldChange | |
| --- | --- | --- | --- | --- |
|  |  |  | RNA-seq | RT-qPCR |
| HSVd-g54 | 2 dpi | LOC101204004 | 3.28 | 2.95 |
|  |  | LOC101216568 | 2.91 | 2.89 |
|  | 14 dpi | LOC101204061 | -4.29 | -5.04 |
|  |  | LOC101205677 | -1.69 | -2.02 |
|  | 28 dpi | LOC101212420 | 0.73 | 1.22 |
|  |  | LOC101213224 | 0.74 | 0.96 |
| HSVd-h | 2 dpi | LOC101208445 | -2.74 | -2.73 |
|  | 14 dpi | LOC101209949 | 3.58 | 3.72 |
|  | 28 dpi | LOC101208068 | 0.46 | 0.01 |
|  |  | LOC101220230 | 0.46 | -0.04 |
