# Supplementary material for: Global Transcriptomic Changes Induced by Infection of Cucumber (Cucumis sativus L.) with Mild and Severe Variants of Hop Stunt Viroid
Source: Front Microbiol. 2017 Dec 12;8:2427. doi: 10.3389/fmicb.2017.02427 (PMC5733102; doi:10.3389/fmicb.2017.02427)
Supplement: Table S7 — Effect of HSVd infection on the expression of cucumber genes involved in the basal defense response. [file Table7.DOC]

Table S7 Effect of HSVd infection on the expression of cucumber genes involved in the basal defense response

| **Sample** | | **Gene ID** | **log2FoldChange**^*^ | **p-adjusted** | **Function** |
| --- | --- | --- | --- | --- | --- |
| HSVd-h | 14 dpi | LOC101211811 | Inf | 2.87E-03 | basic form of pathogenesis-related protein 1-like (PR1) |
|  | 28 dpi | LOC101203560 | 0.70 | 5.16E-04 | cyclic nucleotide gated channel (CNGCs) |
|  |  | LOC101215903 | 0.72 | 1.14E-02 | cyclic nucleotide gated channel (CNGCs) |
|  |  | LOC101221781 | 0.62 | 5.33E-03 | cyclic nucleotide gated channel (CNGCs) |
|  |  | LOC101211964 | 1.26 | 1.12E-05 | calcium-dependent protein kinase (CDPK) |
|  |  | CDPK5 | 0.55 | 2.80E-02 | calcium-dependent protein kinase (CDPK) |
|  |  | LOC101218013 | 0.56 | 1.54E-02 | calcium-dependent protein kinase (CDPK) |
|  |  | LOC101222854 | 0.85 | 5.86E-03 | calcium-dependent protein kinase (CDPK) |
|  |  | LOC101208990 | 0.52 | 3.52E-02 | calcium-dependent protein kinase (CDPK) |
|  |  | LOC101209614 | 0.98 | 7.09E-03 | respiratory burst oxidase (Rboh) |
|  |  | LOC101219117 | 0.57 | 9.27E-03 | calcium-binding protein CML (CaMCML) |
|  |  | LOC101212011 | 4.01 | 5.64E-09 | calcium-binding protein CML (CaMCML) |
|  |  | LOC101220878 | 1.36 | 1.64E-02 | calcium-binding protein CML (CaMCML) |
|  |  | LOC101220738 | 1.65 | 3.21E-13 | calcium-binding protein CML (CaMCML) |
|  |  | LOC101203511 | 2.53 | 1.11E-04 | calcium-binding protein CML (CaMCML) |
|  |  | LOC101207158 | 1.66 | 1.06E-04 | WRKY transcription factor 33 (WRKY26/33) |
|  |  | LOC101205651 | 3.00 | 1.30E-14 | WRKY transcription factor 33 (WRKY26/33) |
|  |  | LOC101216386 | 0.70 | 2.00E-02 | brassinosteroid insensitive 1-associated receptor kinase 1 (BAK1/BKK1) |
|  |  | LOC101211811 | Inf | 6.51E-15 | basic form of pathogenesis-related protein 1-like (PR1) |
|  |  | LOC101211732 | 1.35 | 1.98E-03 | basic form of pathogenesis-related protein 1-like (PR1) |
|  |  | LOC101219669 | 0.60 | 1.37E-02 | mitogen-activated protein kinase kinase 4/5 (MKK4/5) |
| HSVd-g54 | 2 dpi | LOC101221781 | 0.43 | 2.68E-04 | cyclic nucleotide gated channel (CNGCs) |
|  |  | LOC101215161 | 0.45 | 2.63E-05 | calcium-dependent protein kinase (CDPK) |
|  |  | LOC101220131 | 0.99 | 1.61E-05 | calcium-dependent protein kinase (CDPK) |
|  |  | LOC101208990 | 1.67 | 6.84E-35 | calcium-dependent protein kinase (CDPK) |
|  |  | LOC101222854 | 1.65 | 7.37E-59 | calcium-dependent protein kinase (CDPK) |
|  |  | LOC101218270 | 1.19 | 2.39E-21 | calcium-dependent protein kinase (CDPK) |
|  |  | LOC101208369 | 0.49 | 1.92E-03 | calcium-dependent protein kinase (CDPK) |
|  |  | LOC101209614 | 2.04 | 1.08E-14 | respiratory burst oxidase (Rboh) |
|  |  | LOC101209761 | 1.78 | 1.33E-07 | calcium-binding protein CML (CaMCML) |
|  |  | LOC101206440 | 0.55 | 3.22E-03 | calcium-binding protein CML (CaMCML) |
|  |  | LOC101220025 | 2.88 | 6.05E-12 | calcium-binding protein CML (CaMCML) |
|  |  | LOC101203816 | 0.26 | 4.19E-02 | calcium-binding protein CML (CaMCML) |
|  |  | LOC101220738 | 1.84 | 1.19E-17 | calcium-binding protein CML (CaMCML) |
|  |  | LOC101205441 | 0.63 | 8.39E-06 | calcium-binding protein CML (CaMCML) |
|  |  | LOC101207843 | 3.57 | 2.05E-02 | calcium-binding protein CML (CaMCML) |
|  |  | LOC101220878 | 3.14 | 2.76E-04 | calcium-binding protein CML (CaMCML) |
|  |  | LOC101218237 | 0.89 | 3.51E-02 | calcium-binding protein CML (CaMCML) |
|  |  | LOC101220235 | 0.58 | 1.36E-02 | calcium-binding protein CML (CaMCML) |
|  |  | LOC101222462 | 1.02 | 4.16E-02 | calcium-binding protein CML (CaMCML) |
|  |  | LOC101207390 | 0.76 | 1.22E-02 | calcium-binding protein CML (CaMCML) |
|  |  | LOC101210877 | 0.99 | 8.37E-06 | calcium-binding protein CML (CaMCML) |
|  |  | LOC101205651 | 3.37 | 5.08E-09 | WRKY transcription factor 33 (WRKY26/33) |
|  |  | LOC101207158 | 1.86 | 4.21E-04 | WRKY transcription factor 33 (WRKY26/33) |
|  |  | LOC101215973 | 1.95 | 2.13E-08 | LRR receptor-like serine/threonine-protein kinase FLS2 (FLS2) |
|  |  | LOC101218538 | 0.91 | 1.88E-15 | mitogen-activated protein kinase kinase kinase 1 (MEKK1) |
|  |  | LOC101216386 | 0.89 | 4.88E-07 | brassinosteroid insensitive 1-associated receptor kinase 1 (BAK1/BKK1) |
|  | 14 dpi | LOC101215903 | 1.36 | 1.20E-09 | cyclic nucleotide gated channel (CNGCs) |
|  |  | CDPK5 | 0.59 | 9.17E-03 | calcium-dependent protein kinase (CDPK) |
|  |  | LOC101212879 | 0.77 | 3.34E-02 | respiratory burst oxidase (Rboh) |
|  |  | LOC101220878 | 2.67 | 2.54E-08 | calcium-binding protein CML (CaMCML) |
|  |  | LOC101220738 | 0.80 | 2.33E-04 | calcium-binding protein CML (CaMCML) |
|  |  | LOC101211811 | Inf | 4.64E-05 | basic form of pathogenesis-related protein 1-like (PR1) |
|  | 28 dpi | LOC101220878 | 1.76 | 3.55E-03 | calcium-binding protein CML (CaMCML) |
|  |  | LOC101220738 | 1.43 | 4.65E-03 | calcium-binding protein CML (CaMCML) |
|  |  | LOC101212011 | 3.91 | 8.46E-03 | calcium-binding protein CML (CaMCML) |
|  |  | LOC101205651 | 2.51 | 1.37E-05 | WRKY transcription factor 33 (WRKY26/33) |
|  |  | LOC101211811 | Inf | 1.86E-02 | basic form of pathogenesis-related protein 1-like (PR1) |
| HSVd-g54 vs HSVd-h | 2 dpi | LOC101221781 | 0.28 | 2.33E-02 | cyclic nucleotide gated channel (CNGCs) |
|  |  | LOC101215161 | 0.35 | 2.40E-04 | calcium-dependent protein kinase (CDPK) |
|  |  | LOC101220131 | 0.97 | 3.04E-05 | calcium-dependent protein kinase (CDPK) |
|  |  | LOC101208990 | 1.49 | 2.50E-25 | calcium-dependent protein kinase (CDPK) |
|  |  | LOC101222854 | 1.38 | 1.01E-15 | calcium-dependent protein kinase (CDPK) |
|  |  | LOC101218270 | 1.11 | 3.40E-21 | calcium-dependent protein kinase (CDPK) |
|  |  | LOC101208369 | 0.43 | 9.03E-03 | calcium-dependent protein kinase (CDPK) |
|  |  | LOC101209614 | 1.54 | 4.87E-10 | respiratory burst oxidase (Rboh) |
|  |  | LOC101209761 | 1.78 | 1.48E-07 | calcium-binding protein CML (CaMCML) |
|  |  | LOC101206440 | 0.54 | 1.38E-02 | calcium-binding protein CML (CaMCML) |
|  |  | LOC101220025 | 3.01 | 6.15E-14 | calcium-binding protein CML (CaMCML) |
|  |  | LOC101220738 | 1.93 | 1.02E-19 | calcium-binding protein CML (CaMCML) |
|  |  | LOC101205441 | 0.65 | 7.09E-07 | calcium-binding protein CML (CaMCML) |
|  |  | LOC101207843 | 3.52 | 2.21E-02 | calcium-binding protein CML (CaMCML) |
|  |  | LOC101211506 | 1.23 | 7.26E-07 | calcium-binding protein CML (CaMCML) |
|  |  | LOC101220878 | 1.88 | 2.32E-02 | calcium-binding protein CML (CaMCML) |
|  |  | LOC101212685 | 0.81 | 3.58E-02 | calcium-binding protein CML (CaMCML) |
|  |  | LOC101207390 | 0.53 | 4.53E-02 | calcium-binding protein CML (CaMCML) |
|  |  | LOC101210877 | 0.79 | 2.46E-05 | calcium-binding protein CML (CaMCML) |
|  |  | LOC101205651 | 1.97 | 1.22E-03 | WRKY transcription factor 33 (WRKY26/33) |
|  |  | LOC101207158 | 1.03 | 4.75E-02 | WRKY transcription factor 33 (WRKY26/33) |
|  |  | LOC101215973 | 2.43 | 2.62E-11 | LRR receptor-like serine/threonine-protein kinase FLS2 (FLS2) |
|  |  | LOC101218538 | 0.78 | 1.41E-13 | mitogen-activated protein kinase kinase kinase 1 (MEKK1) |
|  |  | LOC101219669 | 0.35 | 3.81E-03 | mitogen-activated protein kinase kinase 5-like (MEKK5) |
|  |  | LOC101216386 | 1.09 | 2.19E-07 | brassinosteroid insensitive 1-associated receptor kinase 1 (BAK1/BKK1) |
|  |  | LOC101207890 | 0.40 | 3.02E-03 | heat shock cognate protein 80-like |
|  |  | LOC101221822 | 0.31 | 2.22E-02 | heat shock cognate protein 80-like |
|  | 14 dpi | LOC101215903 | 1.02 | 2.14E-04 | cyclic nucleotide gated channel (CNGCs) |
|  |  | LOC101218237 | -1.09 | 3.82E-04 | calcium-binding protein CML (CaMCML) |
|  | 28 dpi | LOC101209614 | -1.03 | 9.15E-03 | respiratory burst oxidase (Rboh) |

^*^Inf, Infinite (The denominator is zero.)
