# Supplementary material for: Global Transcriptomic Changes Induced by Infection of Cucumber (Cucumis sativus L.) with Mild and Severe Variants of Hop Stunt Viroid
Source: Front Microbiol. 2017 Dec 12;8:2427. doi: 10.3389/fmicb.2017.02427 (PMC5733102; doi:10.3389/fmicb.2017.02427)
Supplement: Table S8 — KEGG pathway analysis of plant hormone signal transduction in HSVd-infected cucumber. Results for HSVd-h vs. mock, HSVd-g54 vs. mock, and HSVd-g54 vs. HSVd-h comparisons are shown. [file Table8.DOCX]

Table S8 KEGG pathway analysis of plant hormone signal transduction in HSVd-infected cucumber.

| **Sample** | | **Input number** | **Corrected P-Value** | **Plant hormone** |
| --- | --- | --- | --- | --- |
| HSVd-h | 14 dpi | 4 | 1.44E-02 | IAA, ET, JA, SA |
|  | 28 dpi | 57 | 2.72E-03 | IAA, CK, GA, ABA, ET, BR, JA, SA |
| HSVd-g54 | 2 dpi | 35 | 4.29E-05 | IAA, CK, GA, ABA, ET, BR, JA, SA |
|  | 14 dpi | 18 | 3.28E-02 | IAA, CK, GA, ABA, ET, BR, JA, SA |
|  | 28 dpi | 13 | 5.44E-03 | IAA, CK, GA, ABA, ET, SA |
| HSVd-g54 vs HSVd-h | 28 dpi | 4 | 4.96E-03 | ABA |

IAA, Auxin; CK, Cytokinin; GA, Gibberellin; ABA, Abscisic acid; ET, Ethylene; BR, Brassinosteroid; JA, Jasmonic acid; SA, Salicylic acid
